# In Vivo Antioxidant Activity of Common Dietary Flavonoids: Insights from the Yeast Model *Saccharomyces cerevisiae*

**DOI:** 10.3390/antiox13091103

**Published:** 2024-09-12

**Authors:** Graziana Assalve, Paola Lunetti, Vincenzo Zara, Alessandra Ferramosca

**Affiliations:** Department of Experimental Medicine, University of Salento, I-73100 Lecce, Italy; graziana.assalve@unisalento.it (G.A.); paola.lunetti@unisalento.it (P.L.); vincenzo.zara@unisalento.it (V.Z.)

**Keywords:** bioactive molecules, antioxidants, oxidative stress, *S. cerevisiae*

## Abstract

Background: Oxidative stress, associated with diseases and aging, underscores the therapeutic potential of natural antioxidants. Flavonoids, known for scavenging free radicals and modulating cell signaling, offer significant health benefits and contribute to longevity. To explore their in vivo effects, we investigated the antioxidant activity of quercetin, apigenin, luteolin, naringenin, and genistein, using *Saccharomyces cerevisiae* as a model organism. Methods: We performed viability assays to evaluate the effects of these compounds on cell growth, both in the presence and absence of H_2_O_2_. Additional assays, including spot assays, drug drop tests, and colony-forming unit assays, were also conducted. Results: Viability assays indicated that the tested compounds are non-toxic. H_2_O_2_ reduced yeast viability, but flavonoid-treated cells showed increased resistance, confirming their protective effect. Polyphenols scavenged intracellular reactive oxygen species (ROS) and protected cells from oxidative damage. Investigations into defense systems revealed that H_2_O_2_ induced catalase activity and oxidized glutathione accumulation, both of which were reduced by polyphenol treatment. Conclusions: The tested natural compounds enhance cell viability and reduce oxidative damage by scavenging ROS and modulating antioxidant defenses. These results suggest their potential as supplements and pave the way for further research.

## 1. Introduction

Oxygen, a highly reactive molecule, can undergo partial reduction to form reactive oxygen species (ROS) such as superoxide anion (O_2_^•−^), hydroxyl radicals (^•^OH), hydrogen peroxide (H_2_O_2_), and singlet oxygen (^1^O_2_) [1]. While ROS play essential roles in physiological processes like apoptosis, immunity, and cell signaling [2,3,4], an excess of ROS (often triggered by environmental factors such as UV radiation, pollutants, and smoking) can lead to oxidative stress. This imbalance between ROS production and antioxidant defenses results in damage to cellular components like proteins, lipids, and DNA [5], which is linked to various diseases, including metabolic diseases, cancer, neurological and cardiovascular disorders, infertility, and aging [6,7,8,9,10,11,12]. Therefore, reducing oxidative stress is widely recognized as beneficial for human health [13].

Natural antioxidants, particularly those found in plant-derived foods such as fruits and vegetables, are effective in mitigating oxidative stress [14,15,16,17,18,19]. Among these, flavonoids have garnered attention due to their ability to scavenge free radicals, modulate redox signaling, and enhance antioxidant defenses [18,20,21,22].

Key flavonoids studied for their health benefits include quercetin (QRC), apigenin (API), luteolin (LUT), naringenin (NRG), and genistein (GEN) [23,24,25,26,27]. While the antioxidant potential of plant polyphenols has been widely investigated by in vitro analyses, recent studies have highlighted the extensive metabolism (metabolism that accounts for more than 90% of total compound elimination) [28] of phenols in vivo, leading to remarkable changes in their redox potential.

Therefore, it is crucial to investigate these effects in vivo, with simpler model systems like *Saccharomyces cerevisiae* providing valuable insights. Yeast is a genetically tractable organism with rapid growth and similarities to higher eukaryotes, making it an ideal model for preliminary investigations [29,30,31,32,33,34,35,36,37].

In the present study, we used *S. cerevisiae* cells to systematically analyze the effects of selected antioxidant molecules, some of which have been partially tested in the same model (QRC and GEN) [31,35,36], as well as other bioactive molecules that have never been tested. We assessed the compounds’ effects on cell viability and their impact on cell growth under H_2_O_2_-induced stress, and we evaluated oxidative stress markers such as intracellular ROS levels, protein carbonyl content, glutathione levels, and catalase activity.

To our knowledge, this is the first study to systematically investigate the effects of common dietary flavonoids in a yeast model, covering both physiological and molecular levels. By conducting this comprehensive analysis, we aim to elucidate the antioxidant mechanisms of these compounds and assess their potential protective roles against oxidative damage.

## 2. Materials and Methods

### 2.1. Chemicals

All chemicals were purchased from Sigma (St. Louis, MO, USA). Chemicals used in this study were QRC (CAS no. 117-39-5), NRG (CAS no. 67604-48-2), GEN (CAS no. 466-72-0), API (CAS no. 520-36-5), and LUT (CAS no. 491-70-3). Culture media components were purchased from Becton, Dickinson and Company (Franklin Lakes, NJ, USA).

### 2.2. Yeast Strains and Growth Conditions

*S. cerevisiae* wild-type strain BY4742 (MATα, *his3*Δ1, *leu2*Δ0, *lys2*Δ0, *ura3*Δ0) was provided by the EUROFAN resource center EUROSCARF (Frankfurt, Germany).

Yeast cells were grown in YP containing 2% (*w*/*v*) bactopeptone and 1% (*w*/*v*) yeast extract at pH 4.8 or in synthetic complete medium (0.67% *w*/*v* yeast nitrogen base, 0.1% *w*/*v* drop-out mix), at 30 °C. All media were supplemented with a fermentable (2% *w*/*v* glucose) carbon source [38]. For all the experiments, cells were grown in a YPD liquid medium to the exponential phase in an orbital shaker incubator at 30 °C.

### 2.3. Optimization of Bioactives Concentration

The exponentially grown wild-type cells were treated with different concentrations (1–100 μM) of each compound. After incubating for 24 h, the optical cell density (OD_600_) was obtained and the relative growth (%) was expressed as normalized to untreated cells grown in the presence of 10 mM DMSO, the solvent in which the molecules were solubilized. Colony-forming unit (CFU) assays were carried out by spreading the appropriate dilution of the cells on glucose-supplemented SC plates containing 1–100 μM of each compound. After incubating for 24 h, CFUs were counted and expressed as a relative percentage viability compared to the DMSO control (100%).

### 2.4. Assessment of Antioxidant Activity of Bioactive Chemicals

After overnight culturing of wild-type cells in YPD, cells were regrown in a glucose-supplemented SC medium in a 96-well plate and treated with 10 μM of each bioactive for 2 h followed by exposure to H_2_O_2_ (2 mM). The OD_600_ measurements at the indicated time were used to generate the growth curves and to calculate growth parameters such as the doubling time and growth rate. For spot assays, exponentially grown wild-type cells were treated with or without the bioactive compound for 2 h, serially diluted, and spotted on a glucose-supplemented SC plate or a glucose-supplemented SC plate containing H_2_O_2_ (2 mM). The plates were incubated at 30 °C for 2 days. For CFU counts, cells were treated as described before and spread on glucose-supplemented SC plates containing 2 mM H_2_O_2_ in triplicate. After 2 days of incubation, CFUs were counted and expressed as a relative percentage viability compared to the respective control (100%) [39].

### 2.5. Measurement of Oxidative Damage

Exponentially grown wild-type cells were directly stressed in presence of 2 mM H_2_O_2_ for 1 h, or previously treated with the tested compounds (10 μM) during 2 h at 30 °C in a shaker incubator. The cells were harvested by centrifugation for 5 min at 3000× *g* and used for the measurement of intracellular ROS and protein carbonylation.

The ROS levels in the cells were determined by using 2,7-dichlorofluorescein-diacetate (H_2_DCF-DA), which is cleaved by intracellular oxidants to form 2,7-dichlorofluorescein (DCF), a highly fluorescent product. The cells were washed in PBS (phosphate buffer saline, pH 7.4) and incubated with H_2_DCF-DA (20 μM) in the dark for 30 min at 150 rpm and 30 °C. Cells were harvested by centrifugation for 5 min at 3000× *g* and washed twice with PBS buffer. Cells were then centrifuged on ice, and the supernatant was collected in a 96-well plate and observed under a fluorescent microplate reader with an excitation-emission wavelength 485–525 nm [40].

Protein carbonyl content is widely used as a marker for oxidative stress. Carbonylated proteins were detected in the total extract from the yeast cells by using the OxyBlot™ Protein Oxidation Detection Kit (Merck Millipore, Billerica, MA, USA), according to the manufacturer’s instructions. Image analysis was carried out using the ChemiDoc imaging system and Image Lab 6.1.0 software (Bio-Rad Laboratories, Hercules, CA, USA) [41].

### 2.6. Determination of Yeast Antioxidant Capacity

Cells were cultivated and treated as previously described. Measurement of catalase (CAT) activity and determination of oxidized glutathione (GSSG) content were conducted on yeast cell-free extract. Cells, washed twice with KPi containing 0.1 mM PMSF, were suspended in the same buffer, and mixed with an equal volume of acid-washed glass beads. Cell suspensions were vortexed (5 × 15 s cycles), and cell debris was removed by centrifugation at 13,000× *g* for 10 min at 4 °C. According to the Bradford procedure, the protein content was determined using bovine serum albumin (BSA) as a standard [42].

CAT activity was determined by following the consumption of H_2_O_2_. Briefly, 50 μL of extract was added to 1 mL of 7.5 mM H_2_O_2_ in 50 mM KPi buffer (pH 7.0), and H_2_O_2_ decomposition was monitored at 240 nm (ε_240_ = 43.6 M^−1^ cm^−1^). One unit of catalase activity catalyzed the degradation of 1 mmol of H_2_O_2_ per min, and results were presented as the average ± SD catalase activity from three independent cultures (n = 3) [42].

### 2.7. Determination of Oxidized Glutathione Content

The content of glutathione in oxidized form (GSSG) was carried on yeast extract pretreated with 10 mM 2-vinylpyridine [43,44]. An equal volume of cold 2 M HClO_4_ containing 4 mM ethylenediaminetetraacetate (EDTA) was added to 100 μL of the extract and mixed thoroughly. After 15 min incubation on ice, suspensions were centrifuged at 2000× *g* for 5 min and the supernatants were neutralized with 3 M KOH at 0 °C. To 200 μL of neutralized extract, 400 μL of 100 mM KPi buffer pH 8.0 and 10 μL of 10 mM DTNB (5,5′-dithio-bis (2-nitrobenzoic acid)) were added. After 5 min incubation on ice absorbance was measured at 412 nm. The results were expressed as μM GSSG/mg of protein.

## 3. Results

### 3.1. Effects of Selected Flavonoids on Cell Viability

**Figure 1:**
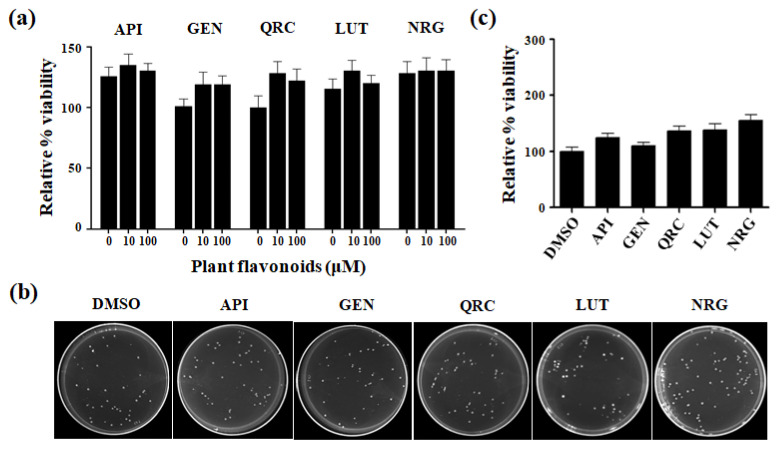
Sensitivity of *S. cerevisiae* cells to selected flavonoids. (**a**) Cells were grown in synthetic complete (SC) medium supplemented with glucose 2% in the presence of different concentrations of bioactive compounds, and the optical cell density (OD_600_) was obtained at 24 h. The relative growth (%) was expressed as normalized to untreated cells grown in the presence of 10 mM DMSO. (**b**) To perform the CFU assay, exponentially grown cells were spread onto glucose-supplemented SC plates containing 10 μM of each compound, the concentration at which cells showed the maximum percentage growth compared to the untreated control. Images were taken 24 h after cell seeding and were representative of three independent experiments. (**c**) The CFU assay data were expressed as a relative percentage of viability, setting the total number of cells in the DMSO control as 100%. Data are mean ± S.D. of three independent experiments.

To evaluate the effects of selected flavonoids on *S. cerevisiae* viability, cells were grown in a YPD liquid medium until the exponential phase and then treated with different concentrations (1–100 μM) of each compound for 24 h. After incubation, the optical cell density (OD_600_) was determined and the relative growth (%) was expressed as the normal value for untreated cells grown in the presence of 10 mM dimethyl sulfoxide (DMSO), the solvent in which the molecules were dissolved (Figure 1a).

Moreover, the exponentially growing cells were diluted and plated onto glucose-supplemented SC plates containing 1–100 μM of each compound to assess CFUs. After a 24 h incubation period, CFUs were counted (Figure 1b) and expressed as a percentage of relative viability compared to the DMSO control (100%) (Figure 1c).

As expected, we found that these compounds were not toxic to the yeast cells within the tested concentration range of 1–100 µM. The cells exhibited over 100% growth compared to the untreated control. Given that the compounds did not affect cell growth, we proceeded to investigate their antioxidant capacity using the minimum concentration (10 μM) at which the maximal percentage growth was observed compared to the untreated control.

### 3.2. Effect of Selected Flavonoids on Oxidative Stress Tolerance

The toxicity of H_2_O_2_ arises from the generation of highly reactive hydroxyl radicals, facilitated by transition metals like iron and copper through the Fenton reaction within cells [45]. Given the pivotal role of H_2_O_2_ in cellular damage and death, we investigated whether the flavonoids used in this study could increase the tolerance of yeast cells to H_2_O_2_.

**Figure 2:**
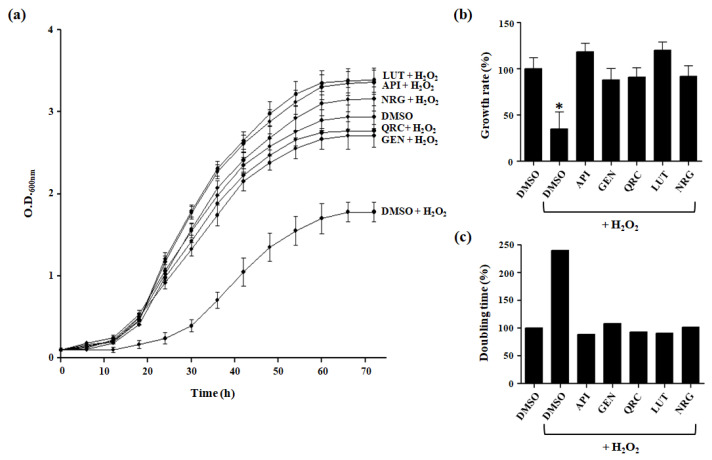
Effect of selected flavonoid treatment on *S. cerevisiae* cell growth. (**a**) Cells were grown with or without tested compounds (10 μM) in liquid glucose-supplemented SC medium containing 2 mM H_2_O_2_. Untreated cells grown in the absence of H_2_O_2_ (DMSO) were used as a control. The optical density values at 600 nm refer to cell cultures after the indicated growth time. Growth curves data are expressed as the mean ± S.D. of three independent experiments. (**b**) Growth rates of yeast cells grown in the glucose-supplemented SC medium with H_2_O_2_ in the presence or absence of the tested compounds were compared to growth rate of yeast cells grown without H_2_O_2_, which was set to 100%. (**c**) The doubling times of yeast cells grown in glucose-supplemented SC medium with H_2_O_2_ in the presence or absence of the tested compounds were compared to the doubling time of yeast cells grown without H_2_O_2_, which was set to 100%. Growth rate data are the mean ± S.D. of three independent experiments, each performed in triplicate. * *p* < 0.005.

Yeast cells, cultured in presence of glucose as carbon source, can undergo fermentation rather than mitochondrial respiration; therefore, ROS levels are reduced. As a consequence, the intracellular antioxidant defense system is repressed, and cells are highly sensitive to oxidative stress. Hence, the exposure to a sublethal concentration of 2 mM H_2_O_2_ resulted in a noticeable decline in the survival rate of the wild-type strain (Figure 2a).

To evaluate the antioxidant potential of the flavonoids, yeast cultures were supplemented with one of the compounds of interest at a concentration of 10 µM, in addition to 2 mM H_2_O_2_, revealing an increased tolerance to oxidative stress. In fact, treatment with each flavonoid led to similar or slightly improved growth compared to DMSO control cells, indicating that these compounds shielded yeast cells from H_2_O_2_-induced cellular toxicity (Figure 2a).

Additionally, to explore a potential relationship between flavonoid treatment and cell growth, we evaluated specific growth rate and doubling time. Under our experimental conditions, cells treated with 10 μM of the tested compounds demonstrated a significant increase in growth rate and a reduction in doubling time, respectively, compared to untreated cells exposed to H_2_O_2_ (Figure 2b,c; Table 1).

To assess the antioxidant capacity of the tested flavonoids, we conducted various growth recovery assays under H_2_O_2_-induced stress, including spot assays, drug drop tests, and CFU assays.

**Figure 3:**
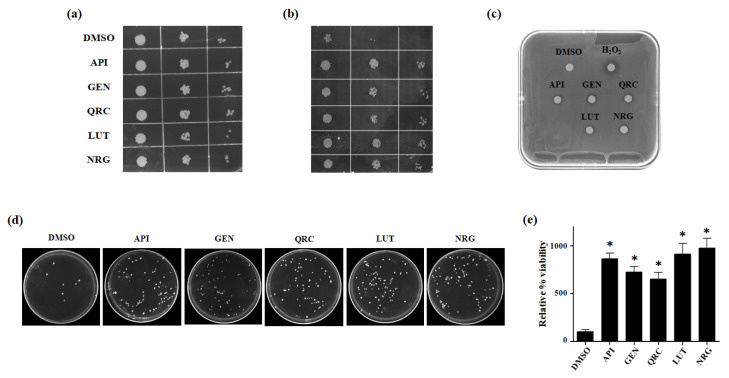
Antioxidant effect of selected flavonoids in *S. cerevisiae*. (**a**) Yeast cells were grown until the exponential phase in the presence of each tested compound (10 μM) or DMSO (10 mM). Then, cells were serially diluted, and spotted onto glucose-supplemented SC control plates, and (**b**) glucose-supplemented SC plates containing 2 mM H_2_O_2_. Plate images were taken at 48 h and are representative of three different experiments. (**c**) 1.5 × 10^6^ cells were spread on a 120 × 120 mm square plate containing solid SC medium supplemented with glucose 2%. Sterile filters were added on the agar surface. Each filter was loaded with 5 μL of one of the tested compounds at the concentration of 10 μM and 5 μL of 2 mM H_2_O_2_. One filter was loaded with the same amount of DMSO without H_2_O_2_. Cell growth was evaluated after 48 h incubation at 30 °C by the halo around the filters. (**d**) Yeast cells were grown with 10 μM of each compound or DMSO for 24 h. Then, an appropriate dilution of the cells was spread onto glucose-supplemented SC plates containing 2 mM H_2_O_2_ to perform CFU assays. Images were representative of three independent experiments. (**e**) CFU assay data were expressed as a relative percentage of viability, setting the total number of cells in DMSO control as 100%. Data are mean ± SD of three independent experiments. * *p* < 0.005.

In the spot assay, which is typically used to assess yeast cell growth on solid media, both flavonoid-treated and untreated cells displayed similar growth patterns on the glucose-supplemented SC medium without H_2_O_2_ (Figure 3a). However, flavonoid-treated cells showed significantly improved survival on glucose agar plates containing 2 mM H_2_O_2_ compared to untreated cells (DMSO) (Figure 3b). The drug drop test further demonstrated enhanced growth in the presence of flavonoids, suggesting that these compounds mitigate the harmful effects of oxidative stress (Figure 3c). Additionally, the observed increase in CFU under stress conditions with polyphenol treatment indicates that more yeast cells survived and reproduced, reinforcing the protective role of flavonoids (Figure 3d,e).

Overall, the combined results from these assays confirm that flavonoids confer a protective effect on yeast cells under oxidative stress, enhancing cell survival and growth.

### 3.3. Effect of Selected Flavonoids on Intracelluar Oxidation

To determine whether the tested flavonoids enhance tolerance to oxidative stress by reducing ROS levels, we measured intracellular oxidation using the fluorescent probe 2′,7′-dichlorofluorescein diacetate (H_2_DCF-DA). This probe is widely used to assess ROS production following oxidative stress, as it becomes fluorescent upon reaction with ROS inside the cell [46].

**Figure 4:**
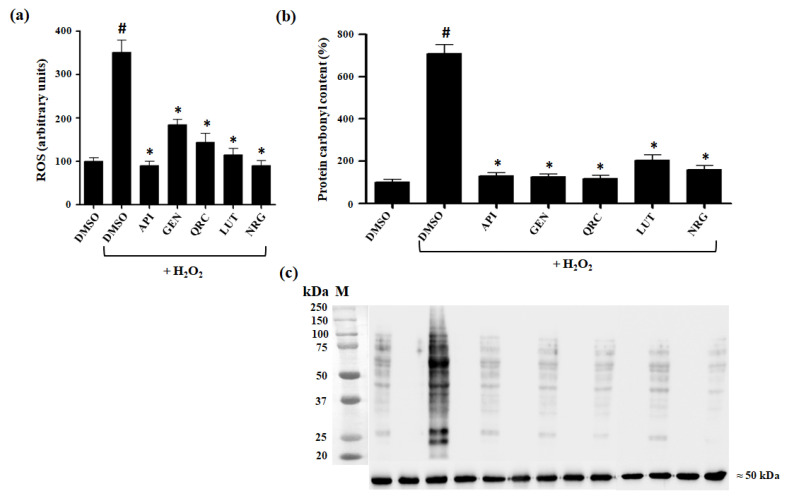
Measurement of oxidative stress markers. (**a**) Quantification of intracellular ROS accumulated during treatment with H_2_O_2_ in yeast cells pretreated with tested polyphenols. At least three independent experiments were performed in triplicate with ^#^ *p* value < 0.001 when compared to DMSO control cells, * *p* value < 0.001 when compared to H_2_O_2_-treated cells in the absence of bioactive compounds. (**b**) Quantification of protein carbonyl content accumulated during treatment with H_2_O_2_ in yeast cells pretreated with tested polyphenols. Densitometry was used for quantitative analysis, with protein carbonyl content in DMSO control cells set to 100%. Data were taken from the same membrane. (**c**) Protein carbonyl content immunodetection with the corresponding control loading (α-tubulin).

Following H_2_O_2_ treatment, a significant increase in intracellular oxidation was observed, reflecting heightened sensitivity to oxidative stress. However, in the presence of one of the tested flavonoids, this increase in ROS levels was notably reversed. Specifically, treatment with each selected flavonoid resulted in nearly a twofold reduction in ROS levels compared to untreated cells, highlighting their strong ROS-scavenging activity (Figure 4a).

Intracellular H_2_O_2_ and its derivatives, such as hydroxyl radicals (·OH) and singlet oxygen (^1^O_2_), are closely associated with cellular damage, affecting membrane components, proteins, and DNA. Reliable biomarkers of oxidative stress are crucial for evaluating cytotoxicity. In addition to measuring intracellular oxidation, protein carbonylation levels are a well-established indicator of oxidative stress. Therefore, to further assess the potential of tested flavonoids in mitigating oxidative damage, we analyzed protein carbonylation levels (Figure 4b).

Exposure of control cells treated with DMSO to H_2_O_2_ resulted in an increase in protein carbonyl content. Remarkably, this oxidative damage induced by H_2_O_2_ was significantly reduced in cells pretreated with the natural compounds (Figure 4b,c).

These findings strongly suggest that these polyphenolic compounds protect cells against H_2_O_2_-induced oxidative damage.

### 3.4. Effect of Selected Flavonoids on the Activity of Antioxidant Defense Systems

In yeast cells, as in higher eukaryotes, the maintenance of intracellular redox balance is tightly regulated by a combination of enzymatic and non-enzymatic defense systems. Among these, free radical scavenging enzymes, such as catalase, serve as the first line of defense against oxidative damage. The secondary defense mechanism relies on non-enzymatic scavengers, with glutathione (GSH) playing a critical role [47].

Catalase is responsible for catalyzing the breakdown of H_2_O_2_ into O_2_ and H_2_O. In *S. cerevisiae*, this function is fulfilled by two enzymes: catalase A and catalase T [48]. Catalase A is primarily involved in breaking down hydrogen peroxide in the peroxisome, thereby protecting cells from the toxic effects of hydrogen peroxide. Catalase T is involved in the detoxification of hydrogen peroxide in the cytoplasm, especially during the stress response [42].

**Figure 5:**
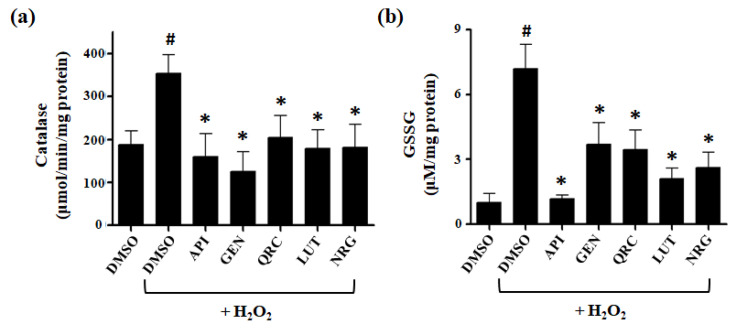
Measurement of antioxidant enzymatic and non-enzymatic defense systems. (**a**) Catalase activity in yeast cells pretreated with different polyphenols (10 µM) for 1 h, followed by incubation with 2 mM H_2_O_2_ for another 1 h. (**b**) GSSG levels in yeast cells pretreated with different polyphenols (10 µM) for 1 h, followed by incubation with 2 mM H_2_O_2_ for another 1 h. The data represent the mean of three independent experiments. ^#^ *p* value < 0.001 when compared to DMSO control cells, * *p* value < 0.001 when compared to H_2_O_2_-treated cells in absence of bioactive compounds.

To explore the potential role of catalase in the protective effects of natural compounds against H_2_O_2_-induced stress, we assessed catalase T activity in yeast cells subjected to various treatments. Yeast cells were treated with DMSO in the presence or absence of H_2_O_2_, or with one of the tested compounds in the presence of H_2_O_2_. A basal level of catalase activity was observed in cells treated with DMSO alone (control). Upon exposure to H_2_O_2_, catalase activity increased, reflecting the enzyme’s response to oxidative stress. Notably, when yeast cells were treated with flavonoids in the presence of H_2_O_2_, catalase activity was reduced to basal levels, suggesting that these compounds may help mitigate intracellular H_2_O_2_ concentrations through a mechanism that does not rely on catalase upregulation (Figure 5a).

GSH is a crucial cellular thiol involved in various cellular processes, including protection against oxidative stress induced by free radicals. H_2_O_2_ triggers the accumulation of oxidized glutathione (GSSG), thereby exacerbating oxidative stress. The non-enzymatic defense mechanism of GSH encompasses a redox-active sulfhydryl group that reacts with oxidants.

To investigate the impact of the flavonoids used in this study on redox homeostasis, we measured oxidized glutathione levels in cells exposed to H_2_O_2_. We observed a sevenfold increase in GSSG levels in response to H_2_O_2_ compared to control cells treated with DMSO. In contrast, cells pretreated with flavonoids showed a significant reduction in H_2_O_2_-induced GSSG accumulation. Specifically, cells pretreated with GEN, QRC, LUT, or NRG exhibited only a two- to threefold increase in GSSG levels after H_2_O_2_ exposure, whereas cells pretreated with API displayed GSSG levels comparable to those of DMSO-treated cells (Figure 5b).

These results are consistent with a reduction in intracellular oxidation and suggest a correlation between the protective effects of natural compounds and the maintenance of redox homeostasis.

## 4. Discussion

Effective regulation of ROS is crucial for maintaining cellular health and longevity, as ROS play complex roles in both physiological processes and pathological conditions. Oxidative stress, resulting from an imbalance between ROS production and antioxidant defenses, is a significant factor in a wide range of acute and chronic diseases, as well as the aging process itself. This has led to increasing interest in natural antioxidants as potential therapeutic agents for oxidative stress-related disorders.

Natural antioxidants, including flavonoids, have garnered attention due to their ability to neutralize free radicals, chelate metal ions, and modulate cellular signaling pathways [25,26]. These compounds are believed to contribute to health benefits by reducing oxidative damage. In fact, diets rich in flavonoid-containing foods have been associated with enhanced longevity and a lower risk of age-related diseases [49].

Despite promising in vitro studies demonstrating the antioxidant properties of these compounds, their in vivo effects remain uncertain. This uncertainty arises from the extensive metabolic transformations these compounds undergo within a living organism, which may modify or diminish their beneficial effects.

To bridge this gap, we investigated the antioxidant activity and potential mechanisms of these compounds in vivo using *S. cerevisiae* as a eukaryotic model organism. This model was selected because it offers a straightforward, systematic, and reproducible approach to assess the antioxidant potential of bioactive molecules with nutritional and therapeutic significance.

In our study, we focused on five flavonoids (QRC, API, LUT, NRG, and GEN), all of which are prevalent in the diet. Prior research has explored the antioxidant and anti-aging potential of QRC and GEN using *S. cerevisiae* as a model, highlighting their beneficial effects [31,35,36]. However, the effects of API, LUT, and NRG in a yeast model have not been previously explored. Additionally, no systematic analysis has compared the effects of these compounds with similar properties from the physiological to molecular levels.

Our objective was to conduct a comprehensive analysis to evaluate the effects of selected compounds, which are known for their health benefits and therapeutic potential, under standardized experimental conditions. Specifically, we aimed to assess yeast cell growth and to elucidate the detailed mechanisms involved in their antioxidant defenses.

Our primary aim was to evaluate the growth phenotype and recovery ability of yeast cells treated with these compounds in the presence of H_2_O_2_, a potent trigger of oxidative stress. To ensure accurate analysis, the yeast cells were cultured in the presence of a fermentable carbon source, thereby excluding ROS that are generated as byproducts of normal respiratory metabolism. This approach allowed us to focus specifically on the effects of exogenously induced oxidative stress on cellular responses.

Our study began by performing viability assays to assess the potential toxicity of the tested compounds. Our results showed that the tested substances did not exhibit toxicity within a certain concentration range. Moreover, in the presence of a sublethal concentration of H_2_O_2_, a significant decrease in the viability of yeast cells was observed. However, the introduction of one of the tested flavonoids into the culture medium restored yeast cell growth to levels comparable to those of non-stressed cells, indicating the protective effect of these polyphenols against oxidative stress. Notably, cells treated with LUT, API, and NRG exhibited improved growth relative to non-stressed cells, although these differences were not statistically significant. The recovery of the growth phenotype was further validated by spot assays, drug drop tests, and CFU assays, underscoring the efficacy of these compounds in promoting cell survival under oxidative stress conditions. These findings suggest that the tested natural compounds confer protection against oxidative stress-induced damage in yeast cells.

The scavenging of intracellular ROS by natural compounds has been identified as a potential mechanism for enhancing cell viability under oxidative stress conditions [20]. In our investigation of flavonoids’ ability to mitigate ROS accumulation and protect yeast cells from oxidative stress-induced damage, we observed a significant reduction in ROS levels, bringing them close to those observed under untreated conditions. This suggests effective cellular protection. Furthermore, treatment with these compounds completely inhibited protein carbonylation induced by H_2_O_2_, providing further confirmation of their antioxidant properties.

*S. cerevisiae* possesses robust antioxidant defense systems, including enzymatic ROS detoxification enzymes such as superoxide dismutase, catalase, and peroxidase, alongside non-enzymatic mechanisms primarily involving GSH [50]. Sublethal doses of H_2_O_2_ trigger a stress response in yeast cells, leading to the upregulation of antioxidant defenses and stress proteins, thereby enhancing cellular resistance to oxidative stress [51]. Notably, the response to H_2_O_2_ in *S. cerevisiae* is closely associated with catalase activity and oxidized glutathione levels [52]. Catalase acts as a potent scavenger of H_2_O_2_, providing critical protection against oxidative damage. Additionally, GSH plays a crucial role in preventing irreversible oxidation of protein cysteine residues, thus preserving cellular integrity [51]. Previous studies have shown that upon exposure to H_2_O_2_, yeast cells induce cytosolic catalase activity and GSH production [42,53,54]. Furthermore, deletion of catalase or glutathione peroxidase 3 (Gpx3) significantly increases sensitivity to H_2_O_2_. Consistent with these findings, our study demonstrated a significant increase in catalase activity following exposure to H_2_O_2_ [42,55]. Interestingly, this increase was not observed in cells pretreated with flavonoids, suggesting that these compounds effectively scavenge ROS through alternative mechanisms. Additionally, exposure to H_2_O_2_ resulted in a notable rise in GSSG levels, indicating heightened oxidative stress and reduced yeast cell viability. However, treatment with flavonoids significantly reduced glutathione oxidation, thereby effectively alleviating cellular stress.

Our results demonstrate that all the tested molecules effectively restore cell viability under stress conditions. This protective effect is largely due to their ability to mitigate oxidative damage by modulating antioxidant defenses. Although the overall protective effects are consistent across the molecules, there are slight differences in their effectiveness. Specifically, the common structural features of the flavonoids tested—particularly the presence of hydroxyl groups and conjugated systems—can be directly related to their antioxidant activity. Variations in structural elements, such as the number and position of hydroxyl groups, can contribute to differences in their antioxidant capacity and overall efficacy.

## 5. Conclusions

This study highlights the significant antioxidant potential of selected flavonoids, as demonstrated using *S. cerevisiae* as a model organism. Our findings reveal that QRC, API, LUT, NRG, and GEN possess notable antioxidant properties. In particular, these molecules were shown to enhance cell viability, reduce oxidative damage, and modulate antioxidant defenses effectively in yeast cells. 

The results suggest that the yeast model is a valuable tool for demonstrating that these natural compounds have potential as dietary supplements or therapeutic agents in mitigating disorders related to oxidative stress. However, further research is essential to assess their in vivo efficacy and to explore their potential applications in health promotion and longevity.

## Figures and Tables

**Table 1:** Growth rate and doubling time of yeast cells grown in glucose-supplemented SC medium with or without H_2_O_2_ in the presence of the tested bioactives. Growth rate data are the mean ± S.D. of three independent experiments, each performed in triplicate. * *p* < 0.005.

		Growth Rate (h^−1^)	Doubling Time (h)
**−H_2_O_2_**	**DMSO**	0.120 ± 0.012	5.78
**+H_2_O_2_**	**DMSO**	0.047 ± 0.015 *	14.61
	**API**	0.143 ± 0.012	4.87
	**GEN**	0.109 ± 0.009	6.37
	**QRC**	0.118 ± 0.011	5.88
	**LUT**	0.145 ± 0.011	4.79
	**NRG**	0.119 ± 0.013	5.82
	**API**	0.143 ± 0.012	4.87

## Data Availability

The datasets generated and analyzed during this current study are available from the corresponding author upon reasonable request.

## References

[1] Li D., Ding Z., Du K., Ye X., Cheng S. (2021). Reactive Oxygen Species as a Link between Antioxidant Pathways and Autophagy. Oxid. Med. Cell Longev..

[2] Chen Y.-R., Zweier J.L. (2014). Cardiac Mitochondria and Reactive Oxygen Species Generation. Circ. Res..

[3] Kasai S., Shimizu S., Tatara Y., Mimura J., Itoh K. (2020). Regulation of Nrf2 by Mitochondrial Reactive Oxygen Species in Physiology and Pathology. Biomolecules.

[4] Sies H., Belousov V.V., Chandel N.S., Davies M.J., Jones D.P., Mann G.E., Murphy M.P., Yamamoto M., Winterbourn C. (2022). Defining Roles of Specific Reactive Oxygen Species (ROS) in Cell Biology and Physiology. Nat. Rev. Mol. Cell Biol..

[5] Pizzino G., Irrera N., Cucinotta M., Pallio G., Mannino F., Arcoraci V., Squadrito F., Altavilla D., Bitto A. (2017). Oxidative Stress: Harms and Benefits for Human Health. Oxid. Med. Cell Longev..

[6] Ferramosca A., Pinto Provenzano S., Montagna D.D., Coppola L., Zara V. (2013). Oxidative Stress Negatively Affects Human Sperm Mitochondrial Respiration. Urology.

[7] Darenskaya M.A., Kolesnikova L.I., Kolesnikov S.I. (2021). Oxidative Stress: Pathogenetic Role in Diabetes Mellitus and Its Complications and Therapeutic Approaches to Correction. Bull. Exp. Biol. Med..

[8] Jelic M., Mandic A., Maricic S., Srdjenovic B. (2021). Oxidative Stress and Its Role in Cancer. J. Cancer Res. Ther..

[9] Shaito A., Aramouni K., Assaf R., Parenti A., Orekhov A., El Yazbi A., Pintus G., Eid A.H. (2022). Oxidative Stress-Induced Endothelial Dysfunction in Cardiovascular Diseases. Front. Biosci. Landmark.

[10] Briyal S., Ranjan A.K., Gulati A. (2023). Oxidative Stress: A Target to Treat Alzheimer’s Disease and Stroke. Neurochem. Int..

[11] Dai X., Hu Y., Jiang L., Lei L., Fu C., Wu S., Zhang X., Zhu L., Zhang F., Chen J. (2023). Decreased Oxidative Stress Response and Oxidant Detoxification of Skin during Aging. Mech. Ageing Dev..

[12] Zara V., Assalve G., Ferramosca A. (2023). Insights into the Malfunctioning of the Mitochondrial Citrate Carrier: Implications for Cell Pathology. Biochim. Biophys. Acta (BBA)-Mol. Basis Dis..

[13] Li Y., Li S., Lin C. (2018). Effect of Resveratrol and Pterostilbene on Aging and Longevity. BioFactors.

[14] Ferramosca A., Di Giacomo M., Zara V. (2017). Antioxidant Dietary Approach in Treatment of Fatty Liver: New Insights and Updates. World J. Gastroenterol..

[15] Nani A., Murtaza B., Sayed Khan A., Khan N.A., Hichami A. (2021). Antioxidant and Anti-Inflammatory Potential of Polyphenols Contained in Mediterranean Diet in Obesity: Molecular Mechanisms. Molecules.

[16] Omidifar N., Moghadami M., Mousavi S.M., Hashemi S.A., Gholami A., Shokripour M., Sohrabi Z. (2021). Trends in Natural Nutrients for Oxidative Stress and Cell Senescence. Oxid. Med. Cell Longev..

[17] Speer H., D’Cunha N.M., Alexopoulos N.I., McKune A.J., Naumovski N. (2020). Anthocyanins and Human Health—A Focus on Oxidative Stress, Inflammation and Disease. Antioxidants.

[18] Di Giacomo M., Zara V., Bergamo P., Ferramosca A. (2020). Crosstalk between Mitochondrial Metabolism and Oxidoreductive Homeostasis: A New Perspective for Understanding the Effects of Bioactive Dietary Compounds. Nutr. Res. Rev..

[19] Ferramosca A., Zara V. (2022). Diet and Male Fertility: The Impact of Nutrients and Antioxidants on Sperm Energetic Metabolism. Int. J. Mol. Sci..

[20] Rajendran M., Manisankar P., Gandhidasan R., Murugesan R. (2004). Free Radicals Scavenging Efficiency of a Few Naturally Occurring Flavonoids: A Comparative Study. J. Agric. Food Chem..

[21] Pandey K.B., Rizvi S.I. (2009). Plant Polyphenols as Dietary Antioxidants in Human Health and Disease. Oxid. Med. Cell Longev..

[22] Ferramosca A., Lorenzetti S., Di Giacomo M., Lunetti P., Murrieri F., Capobianco L., Dolce V., Coppola L., Zara V. (2021). Modulation of Human Sperm Mitochondrial Respiration Efficiency by Plant Polyphenols. Antioxidants.

[23] Salehi B., Fokou P.V.T., Sharifi-Rad M., Zucca P., Pezzani R., Martins N., Sharifi-Rad J. (2019). The Therapeutic Potential of Naringenin: A Review of Clinical Trials. Pharmaceuticals.

[24] Chen H.-I., Hu W.-S., Hung M.-Y., Ou H.-C., Huang S.-H., Hsu P.-T., Day C.-H., Lin K.-H., Viswanadha V.P., Kuo W.-W. (2020). Protective Effects of Luteolin against Oxidative Stress and Mitochondrial Dysfunction in Endothelial Cells. Nutr. Metab. Cardiovasc. Dis..

[25] Kashyap P., Shikha D., Thakur M., Aneja A. (2022). Functionality of Apigenin as a Potent Antioxidant with Emphasis on Bioavailability, Metabolism, Action Mechanism and in Vitro and in Vivo Studies: A Review. J. Food Biochem..

[26] Li Y., Zhang J.-J., Chen R.-J., Chen L., Chen S., Yang X.-F., Min J.-W. (2022). Genistein Mitigates Oxidative Stress and Inflammation by Regulating Nrf2/HO-1 and NF-ΚB Signaling Pathways in Hypoxic-Ischemic Brain Damage in Neonatal Mice. Ann. Transl. Med..

[27] Saberi-Hasanabadi P., Sedaghatnejad R., Mohammadi H. (2024). Protective Effect of Quercetin against Paraquat-Induced Brain Mitochondrial Disruption in Mice. Curr. Drug Saf..

[28] Yuan R., Venitz J. (2000). Effect of Chronic Renal Failure on the Disposition of Highly Hepatically Metabolized Drugs. Int. J. Clin. Pharmacol. Ther..

[29] Katoch B., Begum R. (2003). Biochemical Basis of the High Resistance to Oxidative Stress InDictyostelium Discoideum. J. Biosci..

[30] González Siso M.I., Cerdán M.E. (2012). *Kluyveromyces Lactis*: A Suitable Yeast Model to Study Cellular Defense Mechanisms against Hypoxia-Induced Oxidative Stress. Oxid. Med. Cell Longev..

[31] Bayliak M.M., Burdylyuk N.I., Lushchak V.I. (2016). Quercetin Increases Stress Resistance in the Yeast Saccharomyces Cerevisiae Not Only as an Antioxidant. Ann. Microbiol..

[32] Xing J., Liu P., Zhao L., Huang F. (2017). Deletion of CGLD1 Impairs PSII and Increases Singlet Oxygen Tolerance of Green Alga Chlamydomonas Reinhardtii. Front. Plant Sci..

[33] Agus H.H., Sengoz C.O., Yilmaz S. (2019). Oxidative Stress-Mediated Apoptotic Cell Death Induced by Camphor in *Sod1*-Deficient *Schizosaccharomyces pombe*. Toxicol. Res..

[34] Gao Y., Fang L., Wang X., Lan R., Wang M., Du G., Guan W., Liu J., Brennan M., Guo H. (2019). Antioxidant Activity Evaluation of Dietary Flavonoid Hyperoside Using Saccharomyces Cerevisiae as a Model. Molecules.

[35] Zahoor H., Watchaputi K., Hata J., Pabuprapap W., Suksamrarn A., Chua L.S., Soontorngun N. (2022). Model Yeast as a Versatile Tool to Examine the Antioxidant and Anti-Ageing Potential of Flavonoids, Extracted from Medicinal Plants. Front. Pharmacol..

[36] Gosselin-Monplaisir T., Dagkesamanskaya A., Rigal M., Floch A., Furger C., Martin-Yken H. (2023). A New Role for Yeast Cells in Health and Nutrition: Antioxidant Power Assessment. Int. J. Mol. Sci..

[37] Gröger A., Martínez-Albo I., Albà M.M., Ayté J., Vega M., Hidalgo E. (2023). Comparing Mitochondrial Activity, Oxidative Stress Tolerance, and Longevity of Thirteen Ascomycota Yeast Species. Antioxidants.

[38] Assalve G., Lunetti P., Zara V., Ferramosca A. (2024). Ctp1 and Yhm2: Two Mitochondrial Citrate Transporters to Support Metabolic Flexibility of Saccharomyces Cerevisiae. Int. J. Mol. Sci..

[39] Magistrati M., Gilea A.I., Gerra M.C., Baruffini E., Dallabona C. (2023). Drug Drop Test: How to Quickly Identify Potential Therapeutic Compounds for Mitochondrial Diseases Using Yeast Saccharomyces Cerevisiae. Int. J. Mol. Sci..

[40] Sudharshan S., Veerabhadrappa B., Subramaniyan S., Dyavaiah M. (2019). Astaxanthin Enhances the Longevity of Saccharomyces Cerevisiae by Decreasing Oxidative Stress and Apoptosis. FEMS Yeast Res..

[41] De Blasi G., Lunetti P., Zara V., Ferramosca A. (2024). Mitochondrial Citrate Transporters Ctp1-Yhm2 and Respiratory Chain: A Coordinated Functional Connection in Saccharomyces Cerevisiae Metabolism. Int. J. Biol. Macromol..

[42] Martins D., English A.M. (2014). Catalase Activity Is Stimulated by H_2_O_2_ in Rich Culture Medium and Is Required for H_2_O_2_ Resistance and Adaptation in Yeast. Redox Biol..

[43] Jamnik P., Medved P., Raspor P. (2006). Increased Glutathione Content in YeastSaccharomyces Cerevisiae Exposed to NaCl. Ann. Microbiol..

[44] Owen J.B., Butterfield D.A. (2010). Measurement of Oxidized/Reduced Glutathione Ratio. Methods Mol. Biol..

[45] Zhao Z. (2023). Hydroxyl Radical Generations Form the Physiologically Relevant Fenton-like Reactions. Free Radic. Biol. Med..

[46] Rota C., Fann Y.C., Mason R.P. (1999). Phenoxyl Free Radical Formation during the Oxidation of the Fluorescent Dye 2′,7′-Dichlorofluorescein by Horseradish Peroxidase. J. Biol. Chem..

[47] Fernandes P.N., Mannarino S.C., Silva C.G., Pereira M.D., Panek A.D., Eleutherio E.C.A. (2007). Oxidative Stress Response in Eukaryotes: Effect of Glutathione, Superoxide Dismutase and Catalase on Adaptation to Peroxide and Menadione Stresses in *Saccharomyces cerevisiae*. Redox Rep..

[48] Jamieson D.J. (1998). Oxidative Stress Responses of the Yeast Saccharomyces Cerevisiae. Yeast.

[49] Battino M., Forbes-Hernández T.Y., Gasparrini M., Afrin S., Cianciosi D., Zhang J., Manna P.P., Reboredo-Rodríguez P., Varela Lopez A., Quiles J.L. (2019). Relevance of Functional Foods in the Mediterranean Diet: The Role of Olive Oil, Berries and Honey in the Prevention of Cancer and Cardiovascular Diseases. Crit. Rev. Food Sci. Nutr..

[50] Koleva D.I., Petrova V.Y., Kujumdzieva A.V. (2008). Comparison of Enzymatic Antioxidant Defence Systems in Different Metabolic Types of Yeasts. Can. J. Microbiol..

[51] Costa V., Moradas-Ferreira P. (2001). Oxidative Stress and Signal Transduction in Saccharomyces Cerevisiae: Insights into Ageing, Apoptosis and Diseases. Mol. Aspects Med..

[52] Forman H.J., Zhang H., Rinna A. (2009). Glutathione: Overview of Its Protective Roles, Measurement, and Biosynthesis. Mol. Aspects Med..

[53] Izawa S., Inoue Y., Kimura A. (1995). Oxidative Stress Response in Yeast: Effect of Glutathione on Adaptation to Hydrogen Peroxide Stress in *Saccharomyces cerevisiae*. FEBS Lett..

[54] Penninckx M. (2002). An Overview on Glutathione in versus Non-Conventional Yeasts. FEMS Yeast Res..

[55] Inoue Y., Matsuda T., Sugiyama K., Izawa S., Kimura A. (1999). Genetic Analysis of Glutathione Peroxidase in Oxidative Stress Response of Saccharomyces Cerevisiae. J. Biol. Chem..

